# Pimavanserin and Lorcaserin Attenuate Measures of Binge Eating in Male Sprague-Dawley Rats

**DOI:** 10.3389/fphar.2018.01424

**Published:** 2018-12-07

**Authors:** Amanda E. Price, Victoria D. Brehm, Jonathan D. Hommel, Noelle C. Anastasio, Kathryn A. Cunningham

**Affiliations:** ^1^Center for Addiction Research, University of Texas Medical Branch, Galveston, TX, United States; ^2^Department of Pharmacology and Toxicology, University of Texas Medical Branch, Galveston, TX, United States

**Keywords:** 5-HT_2A_ receptor, 5-HT_2C_ receptor, binge eating, lorcaserin, pimavanserin

## Abstract

Binge eating disorder (BED) is characterized by dysregulated feeding and reward-related processes, and treatment is often challenged by limited therapeutic options. The serotonin (5-HT) 5-HT_2A_ receptor (5-HT_2A_R) and 5-HT_2C_R are implicated in both feeding-related and reward-related behaviors and are thus poised to regulate BED-related behaviors. The purpose of this study was to assess the efficacy of the FDA-approved medications pimavanserin, a 5-HT_2A_R antagonist/inverse agonist, and lorcaserin, a 5-HT_2C_R agonist, in a rodent model of binge eating. The effects of pimavanserin (0.3–3.0 mg/kg), lorcaserin (0.25–1.0 mg/kg), and the lowest effective dose of pimavanserin (0.3 mg/kg) *plus* lorcaserin (1.0 mg/kg) were tested in a high-fat food (HFF) intermittent access binge eating model in adult male Sprague-Dawley rats (*n* = 64). We assessed three measures related to binge eating – binge episode occurrence, binge intake, and weight gain associated with HFF access. Pimavanserin decreased binge intake and weight gain associated with HFF access, but did not prevent binge episode occurrence. Lorcaserin decreased binge intake, but did not prevent binge episode occurrence or weight gain associated with HFF access. Combined pimavanserin *plus* lorcaserin prevented binge episode occurrence in addition to decreasing binge intake and weight gain associated with HFF access. These preclinical findings in male rats suggest that pimavanserin and lorcaserin may be effective in treating patients with BED. Our studies further indicate that administration of one or both drugs may be more effective in certain sub-populations of patients with BED because of the unique profile each treatment elicits. These data support future assessment in clinical populations with BED.

## Introduction

Binge eating disorder (BED) is defined by repeated binge eating episodes that are characterized by uncontrollable, excessive intake of food (American Psyciatric Association, 2013). These episodes are driven by hedonic eating, which can be described as food intake beyond what is physiologically necessary to maintain energy balance (i.e., homeostatic intake of food), and may be caused by disruptions in reward circuitry (Lutter and Nestler, 2009). Current BED treatments in the United States are limited to lisdexamfetamine (Vyvanse^®^), the only FDA-approved pharmaceutical treatment for BED, in addition to behavioral therapy and off-label use of other pharmacological agents (Hutson et al., 2018). We propose that repurposing clinically available drugs that alter both food intake and reward-related behaviors may represent new therapeutic options in the treatment of BED.

Lorcaserin (Belviq^®^) is currently FDA-approved for weight loss in patients with a high body mass index (BMI) and is a viable candidate for drug repurposing in the treatment of BED. Lorcaserin alters both food intake and reward-related processes via activation of the serotonin (5-HT) 5-HT_2C_ receptor (5-HT_2C_R) (for reviews, Higgins et al., 2013; Higgins and Fletcher, 2015). Activation of the 5-HT_2C_R decreases food intake via production of α-melanocyte stimulating hormone, which acts on melanocortin 4 receptors in the paraventricular nucleus of the hypothalamus to promote satiety (Heisler et al., 2002; Xu et al., 2008; Lam et al., 2008). Patients with BED are thought to consume excessive amounts of food in part due to disrupted satiety signals (Sysko et al., 2007), suggesting that satiety signal restoration via administration of a 5-HT_2C_R agonist may decrease food intake during a binge episode. In addition to dysregulated food consumption, people who engage in binge eating also deem palatable foods more rewarding and exhibit greater motivation to consume these substances compared to people who do not binge eat (Finlayson et al., 2011; Dalton et al., 2013; Schebendach et al., 2013). Activation of the 5-HT_2C_R attenuates reward-related behaviors such as drug-taking and drug-seeking (for reviews, Higgins and Fletcher, 2003; Fletcher et al., 2010; Cunningham and Anastasio, 2014; Higgins and Fletcher, 2015) and is therefore likely to dampen hedonic food intake via normalization of reward-related behaviors. Of note, our laboratory and others have demonstrated that selective 5-HT_2C_R agonists decrease binge intake in rodent models (Xu et al., 2017; Price et al., 2018a), further supporting a possible role for lorcaserin in the treatment of BED.

The closely related 5-HT_2A_R also serves as an intriguing target for the treatment of BED. Both the 5-HT_2A_R and 5-HT_2C_R are G-protein coupled receptors that primarily exert effects via G_αq_ signaling pathways. However, these two receptors regulate reward-related behaviors in opposing ways. Specifically, 5-HT_2C_R agonists and 5-HT_2A_R antagonists attenuate reward-related behaviors, while 5-HT_2C_R antagonists promote reward-related behaviors (for review, Cunningham and Anastasio, 2014). Preclinical studies have also indicated that the 5-HT_2A_R is implicated in regulation of feeding behavior. The non-specific 5-HT receptor antagonist metitepine exerts anorectic effects via the 5-HT_2A_R (Gasque et al., 2013), while systemic administration of non-specific 5-HT_2A_R antagonists inhibits overfeeding and obesity in obese A(y) mice and food reinforced operant behavior in fasted Sprague-Dawley rats (Arolfo and McMillen, 1999; Nonogaki et al., 2006). Further, diet-induced obese rats display elevated 5-HT_2A_R binding in the lateral hypothalamus and arcuate nucleus, areas which regulate feeding, [vs. chow fed controls (Park et al., 1999)]. Increased 5-HT_2A_R binding within the nucleus accumbens shell and olfactory nucleus, regions which mediate rewarding effects of food, was observed in diet-induced obese rats or mice relative to controls (Huang et al., 2004; Ratner et al., 2012). Human studies have also demonstrated that BMI positively correlates with *in vivo* cerebral 5-HT_2A_R binding (Erritzoe et al., 2009). Together, these data suggest that 5-HT_2A_R systems are engaged in processes related to food intake. Excitingly, the selective 5-HT_2A_R antagonist/inverse agonist pimavanserin (Nuplazid^®^) is clinically approved for treating psychosis in Parkinson’s Disease and therefore has potential to be repurposed for the treatment of BED.

The present study tested the hypothesis that the clinically available 5-HT_2A_R antagonist/inverse agonist pimavanserin and 5-HT_2C_R agonist lorcaserin decrease parameters related to binge eating. We assessed the effects of both drugs in an intermittent-access high-fat food (HFF) binge eating model in adult male Sprague-Dawley rats on the measures of binge episode occurrence, binge intake, and weight gain associated with HFF exposure. We further tested the hypothesis that combined administration of pimavanserin *plus* lorcaserin would be more effective in decreasing measures related to binge eating than single administration of either drug alone. We chose to assess these three measure related to binge eating to better model clinical studies that assess new possible treatments for BED. While many preclinical studies examine if an intervention can attenuate the amount of food consumed during a binge session (i.e., binge intake), few clinical studies assess this same measure as a primary outcome when evaluating new therapeutics for BED. Instead, clinical studies often use the number of binge episodes in a specific amount of time for different treatment groups as the primary outcome (McElroy et al., 2015). Thus, in addition to measuring food intake during a binge session, we also assessed binge episode occurrence in our preclinical paradigm by dichotomizing whether or not a rat engaged in binge eating behavior (i.e., the rat ate more HFF under intermittent access conditions compared to continuous access conditions) after a given treatment. Further, clinical studies often assess weight change as a secondary outcome. The present study utilized a within-subjects design which limited our assessment of treatment effect on weight gain to only acute effects. Thus, we assessed weight gain during a 22-h period that encompassed drug treatment, HFF exposure, and standard food exposure. The results offer exciting new possibilities in the treatment of BED.

## Materials and Methods

### Animals

Outbred, adult male Sprague-Dawley rats (*n* = 64, Envigo, Haslett, MI) weighing 225-250 g at arrival were single-housed under a 12-h light-dark cycle (lights on between 0600 and 1800 h) with controlled temperature (21–23°C) and humidity (40–50%). Standard food and water were available *ad libitum* except where noted below. Animals were acclimated to the colony room for 7–9 days prior to handling and experimentation. All experiments were conducted in accordance with the NIH *Guide for the Care and Use of Laboratory Animals* (2011) and with the University of Texas Medical Branch Institutional Animal Care and Use Committee approval.

### Food

Standard food chow (Teklad LM-485 Mouse/Rat Sterilizable Diet, Teklad Diets, Madison, WI; 3.1 kcal/g) consisted of 25% protein, 58% carbohydrate, and 17% fat (by kcal). HFF chow (D12451, Research Diets, New Brunswick, NJ; 4.73 kcal/g) contained 20% protein, 35% carbohydrate, and 45% fat (by kcal).

### Drugs

Pimavanserin (0.3, 1.0, or 3.0 mg/mL; Hangzhou Trylead Chemical Technology Co., Ltd., Hangzhou, China) was dissolved in acidic 0.9% NaCl, then brought to a final pH of ∼6.0 using NaOH. Lorcaserin hydrochloride (0.25, 0.5, or 1.0 mg/mL; Hangzhou Trylead Chemical Technology Co., Ltd., Hangzhou, China) was dissolved in 0.9% NaCl. Concentrations were calculated using the salt form of both drugs. All injections were administered subcutaneously at a volume of 1 mL/kg. Pimavanserin and lorcaserin were injected 30 and 15 min prior to behavioral testing, respectively. Doses, routes of administration, and pretreatment times for single administration studies were chosen based on published (Neelakantan et al., 2017; Sholler et al., 2018) studies within our laboratory that suggested these dose ranges affect reward-related behaviors. Doses for the combined administration study were chosen based on the results of the single administration studies (i.e., the lowest dose of drug that decreased binge intake was used).

### Binge Eating Paradigm

The binge eating paradigm used in this study was adapted from a well-established limited access palatable food protocol (Corwin and Wojnicki, 2006; Benzon et al., 2014; Price et al., 2018a,b; and for review, Corwin et al., 2011) and has been validated to induce binge eating behavior in previous publications (Benzon et al., 2014; Price et al., 2018a). The palatable food used in this study was a HFF chow, which is nutritionally representative of foods that patients with BED may eat in excess during a binge episode (Corwin et al., 2011). Rats were given continuous *ad libitum* access to HFF for 7 days to avert food neophobia. On the sixth day of HFF access, 2-h HFF intake was measured in a subset of rats (*n* = 32) in the home cage from 1800–2000 h (beginning of the dark cycle) to determine intake during continuous access conditions. The following day HFF was removed and replaced with standard food which was available *ad libitum* for the remainder of the study except during binge eating sessions. One week after access to HFF, rats began binge eating sessions. Measures related to binge eating were assessed once per week following pharmacological treatment with pimavanserin and/or lorcaserin. On test days, rats received free access to 40 g of HFF in the home cage from 1800–2000 h. At 2000 h, the remaining HFF was removed and weighed, and standard food was made available again.

Three measures related to binge eating were used to assess the effects of drug administration:

#### Binge Episode Occurrence

Binge episode occurrence was assessed to determine if drug treatment could prevent the occurrence of binge eating. The average 2-h HFF intake during continuous access conditions (i.e., on day six of the acclimation period) was set as the minimum intake necessary to constitute a binge episode based on previous studies that have shown that continuous access to HFF does not result in binge eating (Benzon et al., 2014; Price et al., 2018b). This criterion was set using intake as a percent of body weight to control for weight gain throughout the study. Thus, HFF intake more than this percentage during a test session was classified as a binge episode. Rats were dichotomized as exhibiting binge episode occurrence (yes) or not exhibiting binge episode occurrence (no).

#### Binge Intake

Binge intake was assessed to determine if drug treatment could attenuate the magnitude of food consumed during a binge episode. Only rats exhibiting binge episode occurrence after vehicle administration were used to assess this measure. Binge intake was measured in grams of HFF consumed during 2-h access divided by grams of body weight.

#### Weight Gain Associated With HFF Exposure

Weight gain during a 22-h period encompassing drug administration, HFF exposure, and standard food access was analyzed to determine if drug treatment could decrease weight gain associated with exposure to HFF. Rats were weighed at 1400h on the day of the binge, treated with drug between 1730 and 1745 h, given access to HFF from 1800–2000 h, then weighed again at 1200 h the following day. Weight gain was recorded as the difference in body weight in grams from the beginning to the end of this 22-h period.

### Pharmacological Testing

**FIGURE 1:**
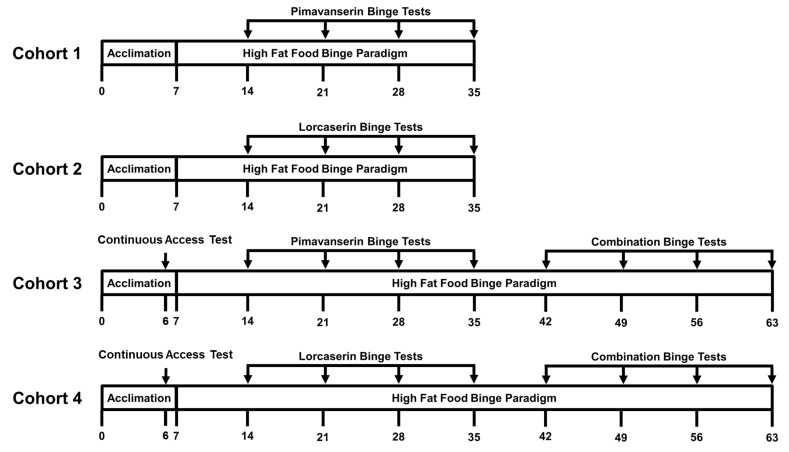
Experimental outline for the assessment of binge eating behaviors. The x-axis indicates day of testing. Acclimation represents a week long period of continuous access to high fat food (HFF) used to prevent food neophobia during binge testing. Two hour HFF intake was measured on Day 6 in Cohorts 3 and 4 to determine HFF intake during non-binge conditions (i.e., during continuous access to HFF).

Four cohorts of rats were used for pharmacological testing (Figure 1). Cohorts 1 (*n* = 16) and 3 (*n* = 16) were injected with vehicle, 0.3, 1.0, or 3.0 mg/kg pimavanserin subcutaneously 30 min prior to the start of the 2-h HFF intake session (1730 h). Cohorts 2 (*n* = 16) and 4 (*n* = 16) were injected with vehicle, 0.25, 0.5, or 1.0 mg/kg lorcaserin subcutaneously 15 min prior to the start of the 2-h HFF intake session (1745 h). Each rat received each dose of the assigned drug in a counterbalanced manner.

After dose response testing was completed, Cohorts 3 and 4 were used to assess the effects of combined administration of pimavanserin *plus* lorcaserin on 2-h HFF intake using the lowest dose of each drug shown to reduce binge intake. Rats were injected with either vehicle or 0.3 mg/kg pimavanserin subcutaneously 30 min prior to the start of the 2-h HFF intake session (1730 h) *plus* vehicle or 1.0 mg/kg lorcaserin subcutaneously 15 min prior to the start of the 2-h HFF intake session (1745 h). Each rat in Cohorts 3 and 4 received each combination of injections in a counterbalanced manner.

### Statistical Analyses

An unpaired Student’s *t*-test was used to ensure the presence of binge eating by comparing HFF intake after continuous access to HFF intake after limited access during vehicle testing. For *binge episode occurrence*, a Cochran’s *Q* test (a non-parametric test that compares differences between three or more sets of binary responses) was used to determine significant differences between drug treatments (Khazaal et al., 2007; Hazra and Gogtay, 2016). *A priori* comparisons were analyzed using multiple McNemar’s tests with a Bonferroni corrected α value of 0.0167 (Cortesi et al., 2004). A Chi-square test was used to determine significant differences in binge episode occurrence after vehicle administration between the four cohorts. Statistical analyses were conducted with an experimentwise error rate of α = 0.05 in SPSS Statistics Version 24. For *binge intake*, a repeated measures one-way analysis of variance (ANOVA) was used to determine significant differences between drug treatments. Subsequent *a priori* comparisons to vehicle were analyzed using Dunnett’s multiple comparisons test. A repeated measures two-way ANOVA was used to assess interactions between treatment with pimavanserin and lorcaserin in the combination study. Statistical analyses were conducted with an experimentwise error rate of α = 0.05 in GraphPad Prism 7. For *weight gain associated with exposure to HFF*, a repeated measures one-way ANOVA was used to determine significant differences between drug treatments. Subsequent *a priori* comparisons to vehicle were analyzed using Dunnett’s multiple comparisons test. Statistical analyses were conducted with an experimentwise error rate of α = 0.05 in GraphPad Prism 7. A two-way mixed model ANOVA using the factors of cohort and treatment was used to assess differences in cohorts between 2-h HFF intake prior to combining the cohorts for analyses. Statistical analyses were conducted with an experimentwise error rate of α = 0.05 in GraphPad Prism 7.

## Results

### Classifying Binge Episode Occurrence

The average 2-h HFF intake after continuous access was 1.47 +/- 0.063% of body weight. Thus, HFF intake of > 1.47% of body weight during test sessions was classified as a binge episode occurrence.

### Effect of Pimavanserin on Binge Episode Occurrence, Binge Intake, and Weight Gain Associated With HFF Exposure

The dose response for pimavanserin on binge episode occurrence, binge intake, and weight gain associated with HFF exposure were assessed in Cohorts 1 and 3. All statistical analyses and results, including from individual and combined cohorts, can be found in Table 1. Statistical analyses for each individual cohort (Cohort 1 and Cohort 3) resulted in consistent conclusions regarding significance for each main effect of treatment assessed. Further, a mixed model two-way ANOVA demonstrated no main effect of cohort at each dose of pimavanserin tested (*F*_1,30_ = 3.368; *p* = 0.0764). Thus, Cohorts 1 and 3 were combined for the analyses presented below to increase achieved power.

**Table 1:** Results from pimavanserin dose-response testing.

	Cohort	Main Effect	Vehicle	0.3 mg/kg	1.0 mg/kg	3.0 mg/kg
Binge episode occurrence^†^	1 (*n* = 16)	*X*^2^_3_ = 1.737; *p* = 0.629	15:1	13:3	15:1	14:2
	3 (*n* = 16)	*X*^2^_3_ = 3.600; *p* = 0.308	14:2	13:3	14:2	11:5
	1+3 (*n* = 32)	*X*^2^_3_ = 3.923; *p* = 0.270	29:3	26:6	29:3	25:7
Binge intake^‡^	1 (*n* = 15)	*F*_3,42_ = 5.159; *p* = 0.004^∗^	0.029 ± 0.002	0.024 ± 0.002; *p* = 0.016^∗^	0.022 ± 0.002; *p* = 0.002^∗^	0.025 ± 0.001; *p* = 0.042^∗^
	3 (*n* = 14)	*F*_3,39_ = 8.443; *p* < 0.001^∗^	0.025 ± 0.002	0.021 ± 0.001; *p* = 0.015^∗^	0.018 ± 0.001; *p* < 0.001^∗^	0.020 ± 0.002; *p* = 0.002^∗^
	1+3 (*n* = 29)	*F*_3,84_ = 12.99; *p* < 0.001^∗^	0.027 ± 0.001	0.022 ± 0.001; *p* < 0.001^∗^	0.020 ± 0.001; *p* < 0.001^∗^	0.022 ± 0.001; *p* < 0.001^∗^
Weight gain associated with HFF exposure^‡^	1 (*n* = 16)	*F*_3,45_ = 7.542; *p* < 0.001^∗^	3.8 ± 0.5	3.3 ± 0.8; *p* = 0.878	1.2 ± 0.5; *p* = 0.008^∗^	0.5 ± 0.6; *p* < 0.001^∗^
	3 (*n* = 16)	*F*_3,45_ = 5.362; *p* = 0.003^∗^	5.3 ± 0.8	4.1 ± 0.7; *p* = 0.502	2.9 ± 0.5; *p* = 0.075	1.2 ± 0.9; *p* = 0.001^∗^
	1+3 (*n* = 32)	*F*_3,93_ = 12.37; *p* < 0.001^∗^	4.6 ± 0.5	3.7 ± 0.5; *p* = 0.412	2.1 ± 0.4; *p* < 0.001^∗^	0.9 ± 0.5; *p* < 0.001^∗^


*^†^Data are represented as the ratio of binge episode occurrence to non-occurrence and were analyzed using a Cochran’s Q test followed by McNemar’s test with a Bonferroni corrected significant α level of 0.0167 (*p*-values are versus vehicle; ^∗^*p* < 0.0167). ^‡^Data are represented as mean ± standard error of the mean and were analyzed using a repeated measures one-way ANOVA followed by Dunnett’s multiple comparisons test (*p*-values are versus vehicle; ^∗^*p* < 0.05).*

**FIGURE 2:**
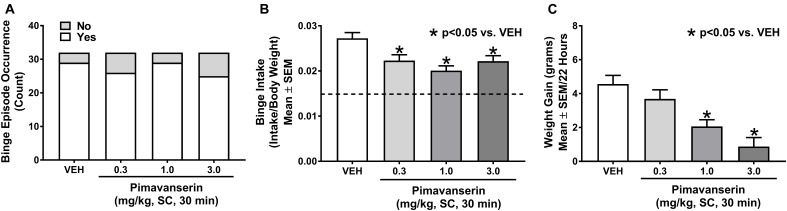
Pimavanserin attenuated binge intake and weight gain associated with high fat food (HFF) exposure but not binge episode occurrence. Pimavanserin (0.3, 1.0, and 3.0 mg/kg) did not alter binge episode occurrence (**A**, *n* = 32), but did decrease binge intake (**B**, *n* = 29). Pimavanserin (1.0 mg/kg and 3.0 mg/kg) reduced weight gain associated with HFF exposure in a dose-related manner (**C**, *n* = 32). Composite data are represented as mean +/– standard error of the mean (SEM). The dashed line on panel **(B)** represents 2-h intake of HFF after continuous access (non-binge intake). ^∗^*p* < 0.05 vs. vehicle (VEH).

An unpaired Student’s *t*-test demonstrated the occurrence of binge eating in the combined Cohort 1 (*n* = 16) and Cohort 3 (*n* = 16) analyses after vehicle treatment (i.e., limited access resulted in a significantly larger intake of HFF compared to continuous HFF access; *p* < 0.001). Cochran’s *Q* test demonstrated no statistically significant difference in binge episode occurrence in the combined Cohort 1 (*n* = 16) and Cohort 3 (*n* = 16) analyses (χ^2^_3_ = 3.923; *p* = 0.270; Figure 2A). Rats exhibiting binge episode occurrence after vehicle administration in Cohorts 1 (*n* = 15) and 3 (*n* = 14) were collapsed into one group; a repeated measures one-way ANOVA revealed a main effect of pimavanserin dose on binge intake (*F*_3,84_ = 12.99; *p* < 0.001). Dunnett’s multiple comparisons test demonstrated that pimavanserin restricted binge intake at 0.3 mg/kg (*p* < 0.001), 1.0 mg/kg (*p* < 0.001), and 3.0 mg/kg (*p* < 0.001) compared to vehicle administration (Figure 2B). This lack of dose-dependent responding is consistent with other studies that have demonstrated that 5-HT_2A_R antagonists often exhibit flat or very narrow dose-response curves on behavioral analyses (Vanover et al., 2006; Nic Dhonnchadha et al., 2009; Anastasio et al., 2011; Sholler et al., 2018). Finally, a repeated measures one-way ANOVA demonstrated a main effect of pimavanserin on weight gain associated with HFF exposure in the combined analyses of Cohort 1 (*n* = 16) and Cohort 3 (*n* = 16) (*F*_3,93_ = 12.37; *p* < 0.001). Dunnett’s multiple comparisons test demonstrated that both 1.0 mg/kg (*p* < 0.001) and 3.0 mg/kg (*p* < 0.001) but not 0.3 mg/kg (*p* = 0.412) pimavanserin significantly decreased weight gain associated with HFF exposure (Figure 2C).

### Effect of Lorcaserin on Binge Episode Occurrence, Binge Intake, and Weight Gain Associated With HFF Exposure

The dose response of lorcaserin on binge episode occurrence, binge intake, and weight gain associated with HFF exposure were assessed in Cohorts 2 and 4. All statistical analyses and results, including from individual and combined cohorts, can be found in Table 2. Statistical analyses for each individual cohort (Cohort 2 and Cohort 4) resulted in consistent conclusions regarding significance for each main effect of treatment assessed. Further, a mixed model two-way ANOVA demonstrated no main effect of cohort at each dose of lorcaserin tested (*F*_1,30_ = 1.111; *p* = 0.3002). Thus, Cohorts 2 and 4 were combined for the analyses presented below to increase achieved power.

**Table 2:** Results from lorcaserin dose-response testing.

	Cohort	Main effect	Vehicle	0.25 mg/kg	0.5 mg/kg	1.0 mg/kg
Binge episode occurrence^†^	2 (*n* = 16)	*X*^2^_3_ = 2.182; *p* = 0.536	13:3	15:1	15:1	13:3
	4 (*n* = 16)	*X*^2^_3_ = 7.444; *p* = 0.059	9:7	14:2	14:2	12:4
	2+4 (*n* = 32)	*X*^2^_3_ = 8.510; *p* = 0.037^∗^	22:10	29:3; *p* = 0.039	29:3; *p* = 0.016^∗^	25:7; *p* = 0.549
Binge intake^‡^	2 (*n* = 13)	*F*_3,36_ = 3.320; *p* = 0.030^∗^	0.025 ± 0.002	0.023 ± 0.002; *p* = 0.717	0.022 ± 0.001; *p* = 0.519	0.019 ± 0.001; *p* = 0.012^∗^
	4 (*n* = 9)	*F*_3,24_ = 7.394; *p* = 0.001^∗^	0.023 ± 0.002	0.026 ± 0.002; *p* = 0.247	0.025 ± 0.002; *p* = 0.489	0.018 ± 0.001; *p* = 0.040^∗^
	2+4 (*n* = 22)	*F*_3,63_ = 7.785; *p* < 0.001^∗^	0.024 ± 0.001	0.024 ± 0.001; *p* = 0.992	0.024 ± 0.001; *p* = 0.987	0.018 ± 0.001; *p* < 0.001^∗^
Weight gain associated with HFF exposure^‡^	2 (*n* = 16)	*F*_3,45_ = 0.7968; *p* = 0.502	4.1 ± 0.8	4.1 ± 0.5	3.3 ± 0.6	2.9 ± 1.0
	4 (*n* = 16)	*F*_3,45_ = 0.5066; *p* = 0.680	3.1 ± 0.9	4.1 ± 1.0	3.6 ± 0.6	2.6 ± 1.0
	2+4 (*n* = 32)	*F*_3,93_ = 0.9926; *p* = 0.400	3.6 ± 0.6	4.1 ± 0.6	3.4 ± 0.4	2.8 ± 0.7


*^†^Data are represented as the ratio of binge episode occurrence to non-occurrence and were analyzed using a Cochran’s Q test followed by McNemar’s test with a Bonferonni corrected significant α level of 0.0167 (*p*-values are versus vehicle; ^∗^*p* < 0.0167). ^‡^Data are represented as mean ± standard error of the mean and were analyzed using a repeated measures one-way ANOVA followed by Dunnett’s multiple comparisons test (*p*-values are versus vehicle; ^∗^*p* < 0.05).*

**FIGURE 3:**
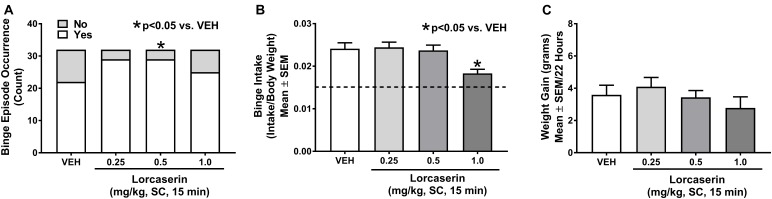
Lorcaserin attenuated binge intake but not binge episode occurrence or weight gain associated with high fat food (HFF) exposure. Lorcaserin (1.0 mg/kg) did not decrease binge episode occurrence (**A**, *n* = 32), but did decrease binge intake (**B**, *n* = 22). Lorcaserin did not alter weight gain associated with HFF exposure (**C**, *n* = 32). Composite data are represented as mean +/– standard error of the mean (SEM). The dashed line on panel **(B)** represents 2-h intake of HFF after continuous access (non-binge intake). ^∗^*p* < 0.05 vs. vehicle (VEH).

An unpaired Student’s *t*-test demonstrated the occurrence of binge eating in the combined Cohort 2 (*n* = 16) and Cohort 4 (*n* = 16) analyses after vehicle treatment (i.e., limited access resulted in a significantly larger intake of HFF compared to continuous HFF access; *p* < 0.001). Cochran’s *Q* test identified a statistically significant difference in binge episode occurrence in the combined Cohort 2 (*n* = 16) and Cohort 4 (*n* = 16) analyses (χ^2^_3_ = 8.510; *p* = 0.037; Figure 3A). Of note, there was no main effect of treatment on binge episode occurrence when Cohort 2 or Cohort 4 was analyzed alone. McNemar’s test was used to identify differences between vehicle and dose using a Bonferroni corrected significant α value of 0.0167. A significant difference between vehicle and 0.5 mg/kg lorcaserin (*p* = 0.016) was identified; however, the analysis indicated that 0.5 mg/kg lorcaserin resulted in a significant *increase* in binge episode occurrence compared to vehicle. This may be due to the low percentage of rats exhibiting binge episode occurrence after vehicle administration in Cohort 4 as a Chi-square test demonstrated a significant difference in binge episode occurrence after vehicle administration between the four cohorts (χ^2^_3_ = 8.012; *p* = 0.046). In Cohorts 1, 2, and 3, greater than 80% of vehicle-treated rats exhibited binge episode occurrence, whereas less than 60% of vehicle-treated rats in Cohort 4 exhibited binge episode occurrence (Tables 1, 2), which may be attributable to environmental and/or genetic factors that contribute to individual differences between outbred rats. Rats exhibiting binge episode occurrence after vehicle administration in Cohorts 2 (*n* = 13) and Cohorts 4 (*n* = 9) were collapsed into one group; a repeated measures one-way ANOVA revealed a main effect of lorcaserin dose on binge intake (*F*_3,63_ = 7.785; *p* < 0.001). Dunnett’s multiple comparisons test demonstrated lorcaserin reduced binge intake at 1.0 mg/kg (*p* < 0.001) compared to vehicle treatment (Figure 3B). Finally, a repeated measures one-way ANOVA demonstrated no main effect of lorcaserin on weight gain associated with HFF exposure when Cohort 2 (*n* = 16) and Cohort 4 (*n* = 16) were combined for analyses (*F*_3,93_ = 0.9926; *p* = 0.400; Figure 3C).

### Effect of Combined Pimavanserin and Lorcaserin on Binge Episode Occurrence, Binge Intake, and Weight Gain Associated With HFF Exposure

Combined administration of effective doses of pimavanserin (0.3 mg/kg) *plus* lorcaserin (1.0 mg/kg) on binge episode occurrence, binge intake, and weight gain associated with HFF exposure were assessed in Cohorts 3 and 4. All statistical analyses and results, including from individual and combined cohorts, can be found in Table 3. Statistical analyses for each individual cohort (Cohort 3 and Cohort 4) resulted in consistent conclusions regarding significance for main effects of treatment observed for binge intake and weight gain associated with HFF exposure. However, Cohort 3 demonstrated only a trend toward a significant main effect of treatment for binge episode occurrence while analyses for Cohort 4 indicated a significant main effect of treatment for this same measure. Further, a mixed model two-way ANOVA demonstrated no main effect of cohort for each treatment tested (*F*_1,30_ = 1.522; *p* = 0.2270). Thus, Cohorts 3 and 4 were combined for the analyses presented below to increase achieved power, but a special discussion that gives possible explanations for the observed differences between cohorts is also included below.

**Table 3:** Results from combined pimavanserin *plus* lorcaserin testing.

	Cohort	Main effect	Vehicle	0.3 mg/kg Pimavanserin Vehicle	Vehicle 1.0 mg/kg Lorcaserin	0.3 mg/kg Pimavanserin 1.0 mg/kg Lorcaserin
Binge episode occurrence^†^	3 (*n* = 16)	*X*^2^_3_ = 7.258; *p* = 0.064	16:0	11:5	11:5	11:5
	4 (*n* = 16)	*X*^2^_3_ = 15.811; *p* = 0.001^∗^	14:2	12:4; *p* = 0.625	8:8; *p* = 0.031	5:11; *p* = 0.004^∗^
	3+4 (*n* = 32)	*X*^2^_3_ = 19.412; *p* < 0.001^∗^	30:2	23:9; *p* = 0.039	19:13; *p* = 0.001^∗^	16:16; *p* < 0.001^∗^
Binge intake^‡^	3 (*n* = 16)	*F*_3,45_ = 4.265; *p* = 0.010^∗^	0.021 ± 0.001	0.020 ± 0.002; *p* = 0.942	0.017 ± 0.001; *p* = 0.014^∗^	0.018 ± 0.001; *p* = 0.042^∗^
	4 (*n* = 14)	*F*_3,39_ = 10.06; *p* < 0.001^∗^	0.022 ± 0.001	0.018 ± 0.001; *p* = 0.070	0.017 ± 0.002; *p* = 0.002^∗^	0.015 ± 0.001; *p* < 0.001^∗^
	3+4 (*n* = 30)	*F*_3,87_ = 12.03; *p* < 0.001^∗^	0.021 ± 0.001	0.020 ± 0.001; *p* = 0.1813	0.017 ± 0.001; *p* < 0.001^∗^	0.016 ± 0.001; *p* < 0.001^∗^
Weight gain associated with HFF exposure^‡^	3 (*n* = 16)	*F*_3,45_ = 4.545; *p* = 0.007^∗^	5.1 ± 0.7	4.0 ± 0.9; *p* = 0.598	2.5 ± 0.8; *p* = 0.050^∗^	1.4 ± 0.9; *p* = 0.004^∗^
	4 (*n* = 16)	*F*_3,45_ = 5.085; *p* = 0.004^∗^	5.1 ± 0.7	3.2 ± 0.8; *p* = 0.239	2.4 ± 0.8; *p* = 0.055	0.8 ± 0.7; *p* = 0.001^∗^
	3+4 (*n* = 32)	*F*_3,93_ = 9.81; *p* < 0.001^∗^	5.1 ± 0.5	3.6 ± 0.6; *p* = 0.133	2.5 ± 0.6; *p* = 0.002^∗^	1.1 ± 0.6; *p* < 0.001^∗^


*^†^Data are represented as the ratio of binge episode occurrence to non-occurrence and were analyzed using a Cochran’s Q test followed by McNemar’s test with a Bonferonni corrected significant α level of 0.0167 (*p*-values are versus vehicle; ^∗^*p* < 0.0167). ^‡^Data are represented as mean ± standard error of the mean and were analyzed using a repeated measures one-way ANOVA followed by Dunnett’s multiple comparisons test (*p*-values are versus vehicle; ^∗^*p* < 0.05).*

**FIGURE 4:**
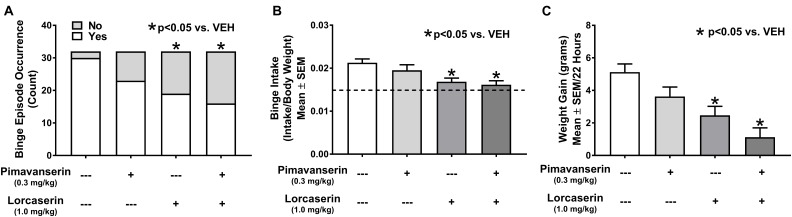
Combined pimavanserin *plus* lorcaserin attenuated binge episode occurrence, binge intake, and weight gain associated with high fat food (HFF) exposure. Both lorcaserin (1.0 mg/kg) alone and pimavanserin (0.3 mg/kg) *plus* lorcaserin (1.0 mg/kg) decreased binge episode occurrence (**A**, *n* = 32), binge intake (**B**, *n* = 25), and weight gain associated with HFF exposure (**C**, *n* = 32). Composite data are represented as mean +/– standard error of the mean (SEM). The dashed line on panel **(B)** represents 2-h intake of HFF after continuous access (non-binge intake). ^∗^*p* < 0.05 vs. vehicle (VEH).

An unpaired Student’s *t*-test demonstrated the occurrence of binge eating in the combined Cohort 3 (*n* = 16) and Cohort 4 (*n* = 16) analyses after vehicle treatment (i.e., limited access resulted in a significantly larger intake of HFF compared to continuous HFF access; *p* < 0.001). Cochran’s *Q* test demonstrated a statistically significant difference in binge episode occurrence in the combined analyses of Cohort 3 (*n* = 16) and Cohort 4 (*n* = 16) (χ^2^_3_ = 19.412; *p* < 0.001; Figure 4A). McNemar’s test (with a Bonferroni corrected significant α value of 0.0167) demonstrated a significant difference in binge episode occurrence after administration of lorcaserin alone (*p* = 0.001) and pimavanserin *plus* lorcaserin (*p* < 0.001) but only a trend for pimavanserin alone (*p* = 0.039). Rats exhibiting binge episode occurrence after vehicle administration in Cohorts 3 (*n* = 16) and Cohorts 4 (*n* = 14) were combined for analyses; a repeated measures one-way ANOVA revealed a main effect of treatment on binge intake (*F*_3,87_ = 12.03; *p* < 0.001). Dunnett’s multiple comparisons test demonstrated a significant decrease in binge intake after administration of lorcaserin alone (*p* < 0.001) and pimavanserin *plus* lorcaserin (*p* < 0.001) but not after administration of pimavanserin alone (*p* = 0.1813; Figure 4B). Finally, a repeated measures one-way ANOVA demonstrated a main effect of treatment on weight gain associated with HFF exposure in combined analyses of Cohort 3 (*n* = 16) and Cohort 4 (*n* = 16) (*F*_3,93_ = 9.81; *p* < 0.0001). Dunnett’s multiple comparisons test demonstrated reduced weight gain associated with HFF exposure following treatment with lorcaserin (*p* = 0.002) and after pimavanserin *plus* lorcaserin (*p* < 0.001; Figure 4C), but not after pimavanserin alone (*p* = 0.133).

The goal of the combination study was to determine if combined administration of pimavanserin and lorcaserin differentially altered measures of binge eating when compared to administration of pimavanserin or lorcaserin alone. Interestingly, 0.3 mg/kg pimavanserin significantly decreased binge intake in the single drug dose-response study, but not in the combination study. Furthermore, 1.0 mg/kg lorcaserin significantly decreased both binge episode occurrence and weight gain associated with HFF exposure in the combination study, but not in the single drug dose-response study. These results were surprising since the single drug dose-response assessments demonstrated consistent results across two independent cohorts. We suspected a possible interaction between drug treatments, so we further analyzed the data in a repeated measures two-way ANOVA using the factors of treatment 1 (pimavanserin) and treatment 2 (lorcaserin). There was a main effect of lorcaserin (*F*_1,29_ = 28.14; *p* < 0.001), but not of pimavanserin (*F*_1,29_ = 2.44; *p* = 0.129), nor a pimavanserin x lorcaserin interaction (*F*_1,29_ = 0.6671; *p* = 0.421).

## Discussion

The present study demonstrated that the selective 5-HT_2A_R antagonist/inverse agonist pimavanserin and selective 5-HT_2C_R agonist lorcaserin are effective at decreasing the magnitude, but not the occurrence, of binge episodes in adult male Sprague-Dawley rats. Pimavanserin, but not lorcaserin, was also effective at restricting weight gain associated with HFF exposure but only at higher doses, thus suggesting a reduction in binge intake alone is not sufficient to decrease weight gain associated with HFF exposure. Excitingly, combined administration of pimavanserin and lorcaserin was effective at decreasing both the occurrence and magnitude of binge episodes in addition to weight gain associated with HFF exposure. These data support future studies assessing the repurposing of these medications for treatment of BED.

Activation of the 5-HT_2C_R attenuates food intake and reward-related behaviors, which in part led to the approval of lorcaserin for weight loss. Our finding that this 5-HT_2C_R agonist reduced binge intake coalesces with our previous results with the selective 5-HT_2C_R agonist WAY163909 (Price et al., 2018a). These findings also align with a recent study demonstrating that lorcaserin decreased binge-like eating in mice via activation of the 5-HT_2C_R localized in dopaminergic neurons (Xu et al., 2017), as well as with studies indicating that 5-HT_2C_R agonists inhibited palatable food intake in non-food-deprived rats (Rowland et al., 2008; Canal et al., 2014). Surprisingly, we did not see an effect of lorcaserin on weight change associated with HFF exposure when tested in the lorcaserin alone study. However, lorcaserin alone decreased weight gain when tested in the combined pimavanserin *plus* lorcaserin study. These results suggest that chronic treatment of lorcaserin may be needed to alter weight gain associated with HFF exposure. This is consistent with previous studies that indicated the effect of lorcaserin on cumulative food intake was time-dependent (Higgins et al., 2015). Since an estimated 70% of people with BED also have elevated BMI (Kessler et al., 2013), identification of a clinically available drug that both restricts binge eating and promotes weight loss would be extremely valuable in this patient population. The anti-obesity medication lorcaserin provides an exciting opportunity as it is currently approved for weight loss, and cumulative evidence suggests its efficacy in treating BED.

The role of the 5-HT_2A_R in feeding-related behaviors is less clear than the role of the 5-HT_2C_R. The present findings agree with previous studies that demonstrated systemic administration of non-specific 5-HT_2A_R antagonists attenuate feeding (Arolfo and McMillen, 1999; Nonogaki et al., 2006; Gasque et al., 2013). Other studies suggest that 5-HT_2A_R DNA hypermethylation, which would be predicted to result in gene inactivation, associates with obesity-related measures (Perez-Cornago et al., 2014) while 5-HT_2A_R agonist administration into the hypothalamus attenuates feeding (Grignaschi et al., 1996; Martin-Gronert et al., 2016), suggesting that a number of pharmacological, genetic, and biochemical factors may contribute to 5-HT_2A_R-mediated feeding-related behaviors. One proposed hypothesis for these discordant findings is that peripherally and centrally expressed 5-HT_2A_R regulate food intake differently (Anderberg et al., 2017), although to our knowledge this hypothesis has not been directly explored. Alternatively, the centrally expressed 5-HT_2A_R may mediate feeding behaviors differently when activated or antagonized in various brain regions. Conversely, the role of the 5-HT_2A_R in reward-related behaviors has been well-studied in the drug addiction field. For example, 5-HT_2A_R blockade reduces reward-seeking behaviors for cocaine, nicotine, and (±)-3,4-methylenedioxymethamphetamine (MDMA) (Orejarena et al., 2011; Fletcher et al., 2012 and for review, Cunningham and Anastasio, 2014). However, 5-HT_2A_R blockade is not effective in reducing self-administration of cocaine or nicotine (Fletcher et al., 2002, 2012; Nic Dhonnchadha et al., 2009 and for review, Cunningham and Anastasio, 2014), suggesting that the 5-HT_2A_R is not directly responsible for mediating drug reward-taking behaviors. While studies have demonstrated overlapping neural mechanisms responsible for driving food-reward and drug-reward behaviors (Volkow et al., 2013), to our knowledge, blockade of the 5-HT_2A_R has not been assessed in operant conditioning paradigms that use HFF pellets as a reinforcer; thus, the specific mechanisms driving 5-HT_2A_R-mediated control of binge eating are still unknown.

Recent studies demonstrate that combinations of pharmacotherapies may be more effective at treating dysregulated eating (e.g., in patients who are overweight or obese who are attempting to lose weight) than monotherapy alone. For example, the weight loss drug Contrave^TM^ (a combined formulation of naltrexone and extended-release bupropion) results in weight loss greater than either drug alone (Greenway et al., 2009). While both single and combined administration of pimavanserin and/or lorcaserin decreased the magnitude of binge episodes, the occurrence of binge episodes was decreased only in the combination study, indicating that pimavanserin and lorcaserin may have behavior-specific interactions. Thus, while single treatments may reduce food consumption during a binge episode in BED, combined therapy may be necessary to prevent the occurrence of binge episodes. This finding is in line with previous preclinical studies that have demonstrated that 5-HT_2A_R antagonists/inverse agonists and 5-HT_2C_R agonists can have additive or even synergistic effects on impulsivity and reward-related behaviors (Cunningham et al., 2013). The present study further supports the ability of combined administration of pimavanserin and lorcaserin to decrease binge episode occurrence, an effect which neither drug alone accomplished, and supports the use of dual therapy or development of combined formulations or heterobivalent ligands to alter reward-related or feeding behaviors, especially the occurrence of binge episodes.

Interestingly, the effects of pimavanserin or lorcaserin alone in the combination study differed from the effects seen in the single drug dose-response study, despite the single drug dose-response study being independently replicated. A two-way ANOVA demonstrated that there is no interaction between pimavanserin and lorcaserin when given concurrently, which suggests that the two drugs are acting independently to reduce intake of HFF, possibly by acting in different brain regions (e.g., pimavanserin may block the 5-HT_2A_R in the lateral hypothalamus and arcuate nucleus while lorcaserin may activate the 5-HT_2C_R in the paraventricular nucleus) (Park et al., 1999; Heisler et al., 2002; Xu et al., 2008; Lam et al., 2008; Canal et al., 2014). However, the possibility that previous exposure to one drug affects subsequent response to the second drug still remains. Other potential explanations for the seemingly discrepant results include altered responses due to repeated exposure of the drug and differences in basal behavior prior to HFF access (e.g., higher stress levels of the rats due to increased number of injections in the combination study). Isobolographic analyses would give further insight into how the two drugs may be interacting to produce behavioral effects.

The different response profiles of pimavanserin, lorcaserin, and the combination of drugs offers the opportunity for individualized treatment for patients with BED. For example, healthy-weight patients who engage in infrequent, but severe, binge episodes may benefit from low-dose pimavanserin since this drug is effective in reducing the magnitude of a binge episode, but does not affect weight change. Conversely, a patient with obesity who also engages in infrequent, but very severe, binge episodes and displays dysregulated eating behaviors beyond BED may benefit most from treatment with lorcaserin since this medication decreased binge magnitude and is also clinically approved for weight loss. Finally, a patient with obesity who experiences binge episodes that are both severe and frequent may benefit most from combined administration of pimavanserin and lorcaserin since this approach prevented binge episode occurrence and decreased both binge magnitude and weight gain associated with exposure to HFF.

The present study gives insight into the potential use of pimavanserin and lorcaserin as therapeutics in the treatment of BED. Future studies are needed to assess the mechanisms by which these drugs exert the observed effects. For example, the binge model employed here does not inform whether the effects observed are specific to hedonic intake or generalizable to homeostatic intake of food. However, binge eating is a complex behavior that is often driven by both hunger and the urge to engage in hedonic eating (Vanderlinden et al., 2001). The timing of the HFF intake evaluations in this study coincides with light-dark cycle switching, which is the time at which rats typically engage in homeostatic intake of food (Spiteri, 1982). Thus, while the paradigm employed herein cannot conclusively separate these two types of eating behaviors, we propose that this experimental design increases the construct validity of the model as the timing of the binge sessions allows for the measurement of intake which may be driven by both homeostatic and hedonic factors. To tease these two mechanisms apart, future studies are needed to assess the efficacy of these medications in both the presence and absence of a negative energy balance (Lowe and Butryn, 2007). In addition, the present study did not investigate whether pimavanserin and lorcaserin specifically decrease HFF intake or if these effects are generalizable to standard food intake. While previous studies have demonstrated that lorcaserin blunts standard food intake (Smith et al., 2008; Thomsen et al., 2008; Fletcher et al., 2009), pimavanserin has been little studied in this regard. Another possible mechanism in which pimavanserin and lorcaserin may act to decrease measures of binge eating is through alteration of stress responses or anxiety-like behavior, both processes which involve these two receptors (Weisstaub et al., 2006; Di Giovanni and De Deurwaerdere, 2016). Of note, it is unlikely that general motor depression accounts for the observed effects as the medication dose ranges employed here do not alter spontaneous motor activity in Sprague-Dawley rats (Levin et al., 2011; Hubbard et al., 2013).

In conclusion, we described two clinically available drugs that have the potential to be successfully repurposed for treatment of BED. Future studies are required to investigate the neurobiological mechanisms underlying the observed effects of these medications. In addition, clinical studies to investigate the safety and efficacy of pimavanserin and/or lorcaserin in the treatment of BED could open the door for new therapeutic options in this population.

## Author Contributions

AP, VB, NA, and KC planned the experiments. JH established the model. AP and VB completed the experiments, analyzed the data, and interpreted the results. AP and VB drafted the manuscript. All authors edited and approved the manuscript.

## Conflict of Interest Statement

The authors declare that the research was conducted in the absence of any commercial or financial relationships that could be construed as a potential conflict of interest.
